# End-of-life care in South Africa: Important legal developments

**DOI:** 10.4102/sajpsychiatry.v28i0.1748

**Published:** 2022-01-18

**Authors:** Carla Kotzé, Johannes L. Roos

**Affiliations:** 1 Department of Psychiatry, Faculty of Health Sciences, University of Pretoria, Pretoria, South Africa; 2 Weskoppies Psychiatric Hospital, Pretoria, South Africa

## Background

All healthcare practitioners should be aware of ethical principles and legislation relevant to end-of-life care. The aim of this scientific letter is to highlight some important South African legal developments that might be especially important for practitioners taking care of vulnerable populations, such as older patients with serious mental illness. A recent unpublished descriptive, cross-sectional, observational study that was conducted at Weskoppies Psychiatric Hospital, in Gauteng, South Africa, found that two thirds of 100 participants older than 60 years of age and diagnosed with a serious mental illness had end-of-life decision-making capacity. This highlights the importance of initiating advance care discussion with this vulnerable population. With the ongoing and important legal developments in South Africa, this population should not be excluded from access to humane end-of-life care that is in keeping with their preferences and values.

## Euthanasia and non-beneficial treatments

The word euthanasia means ‘good death’ and has remained one of the most contentious ethical dilemmas in medical practice around the world.^1^ The issue of assisted voluntary euthanasia was brought to the attention of the South African public with the judgement in *Stransham-Ford v. the Minister of Justice and Correctional Services and Others* (2015), Case no. 27401/15. Judge Fabricius of the North Gauteng High Court found that terminally ill Robin Stransham-applicant had a constitutionally protected right to die with dignity.^2^ This case was opposed by the Minister of Health and the Health Professions Council of South Africa (HPCSA).^2^ Jordaan explained that ‘The legal arguments of both sides centred on constitutional rights – in particular, the right to human dignity, the right to life and the right to control one’s body’.^3^ Human dignity is a very nuanced concept that means different things to different people, but it is an attribute inherent in every human being that should be respected.^3,4^ In the judgement of this case, more weight was given to the right to dignity than to the right to life.^4^

It then transpired that the applicant passed away a few hours before the judgement, and this was decisive in the subsequent appeal. The Supreme Court of Appeal set aside the judgement by the High Court, but there was no engagement with arguments for or against euthanasia. The Supreme Court’s decision was that there was no purpose in granting the order and that the High Court should not have adjudicated the matter after Mr Stransham-Ford’s death.^3^ The Supreme Court did leave the door open for similar future applications to the court, as it was also concluded that assisted suicide is not unlawful in all circumstances. Any future applications will have to be considered on the individual facts, within the context of the *Constitution of the Republic of South Africa,* No. 108 of 1996, which protects the right to life and human dignity.^5^

The debate about euthanasia made headlines again as reported in various South African newspapers during 2019 when Prof. Sean Davison was charged with three counts of murder. He received a suspended sentence of 8 years with house arrest and community service after a court-approved plea was reached in the Western Cape High Court. The compassionate motivation in assisting those people with a dignified death, their requests for his assistance in dying, supported by their relatives and his remorse were all considered as mitigating factors. This case came 44 years after a similar case in 1975 where Dr Hartman assisted the death of his 87-year-old father with terminal cancer. He also received a suspended prison sentence, and it is clear from the similarities between these two cases that there has not been any significant progress in the legal developments on euthanasia since 1975.^6^ Legislation on assisted suicide has previously been developed and proposed but not promulgated, and currently, it is still unlawful in South Africa. Project 86 of the South African Law Commission issued a report in November 1998 titled ‘*Euthanasia and artificial preservation of life*’. This draft bill of the South African Law Commission is a legislative proposal to regulate end-of-life decisions and related matters. The commission did not make any specific recommendations about voluntary active euthanasia but set out different options to get legal clarification on how to deal with this issue. This legislation has yet to be finalised despite tremendous legal and advocacy efforts.^7^

Very recently, a new case was reported in the news that involves a palliative care specialist and one of her patients, both with terminal conditions. They have approached the High Court in Johannesburg, South Africa, to request that the law should be developed to allow for physician-assisted suicide and euthanasia. This case will only start with the first arguments, and evidence from various international experts during 2021 and the outcome and potential influence on the laws in South Africa will not be known in the foreseeable future.^8^ Withholding and withdrawing treatment are considered passive euthanasia and remain an unresolved legal issue locally. In general, it is considered to be permissible and in certain circumstances, even mandatory. In patients where further treatments are considered to be futile or non-beneficial, palliative care should focus on comfort and the patient’s quality of life. There is a perception that death means treatment failure, and this continues to drive the medicalisation of death, prolongation of patient suffering and prevention of high-quality care. It should be kept in mind that it is the disease process that causes death, not the doctor or the treatment that is withdrawn or withheld.^9^

There are many reasons to avoid the excessive use of non-beneficial treatment in patients with life-limiting conditions, including the equitable and sensible use of scarce resources, the avoidance of staff demoralisation when poor outcomes are anticipated and also the avoidance of creating a false sense of hope in patients and families. Non-beneficial or futile treatment can be seen as aggressive management beyond keeping a patient comfortable in situations where the clinical condition of the patient should have prompted a transition to palliative care. The culture of ‘doing everything possible’ has implications for the sustainability of health services. It also perpetuates the unrealistically high societal expectations of survival at all cost, and it disregards human dignity and quality end-of-life care.^10^ To minimise psychological distress for healthcare providers and patients during the coronavirus disease 2019 (COVID-19) pandemic, there have been recommendations for early transition to palliative care, even in emergency departments. This can assist with difficult decisions about rationing of care.^11^

## Living will or advance directives

According to the *National Health Act* No. 61 of 2003 Section 7(1), health services may not be provided to a patient without informed consent. It is implied that if a patient has a living will that declines healthcare, it must be taken into account and treatment should not be provided without the patient’s informed consent. It can be very challenging in cases where there is discordance between the patient’s wishes and those of the family, especially if there is no advance directive.^1^ Any decisions about withholding or withdrawing treatment should take into consideration the values of the patient and their families and the ethical guidelines of the HPCSA. These guidelines make it clear that discrimination based on ‘age, disability, race, colour, culture, beliefs, sexuality, gender, lifestyle, social or economic status or other irrational grounds’ will not be tolerated.^12^

The legality of a living will remains uncertain in South Africa. A notice of intent to introduce a draft Bill, the *National Health Amendment Bill*, was published in the Government Gazette in July 2018. The aim of this Bill is to provide legal recognition, legal certainty and legal enforceability regarding advance healthcare directives such as living wills and durable power of attorney for healthcare. With a durable power of attorney, a substitute decision maker is selected by the patient whilst they can make competent decisions, to act on their behalf, should the patient become incompetent. The proposed legislation has certain limitations, and there is always the possibility of conflicts arising even with advance directives in place, but one of the aims is to provide for the resolution of disputes related to these directives.^13^ This Bill was introduced to the National Assembly for consideration as insertion of sections 7A and 7B into the *National Health Act* No. 61 of 2003, Amendment No. 41789. This amendment can assure patients that their wishes will be carried out and provide legal certainty to doctors, but since its introduction to parliament it has lapsed and it is uncertain if it will be revived.^14^

With all these ongoing legal developments, it is important to emphasise the role of health professionals in the assessment of decision-making capacity, optimisation of decision-making and initiation of advance care discussions. End-of-life discussions should also be initiated with older patients with serious mental illness and cognitive impairments to ensure that all patients are provided with an opportunity to access humane end-of-life care. This will allow for patient preferences to be accommodated within the limits of ethical and legal guidelines, even at times when they no longer have the capacity to express their wishes.
